# Histopathological findings in gallbladders of asymptomatic living liver donors at a liver transplant center

**DOI:** 10.12669/pjms.42.3.13528

**Published:** 2026-03

**Authors:** Haider Ali, Ihsan ul Haq, Sohail Rashid, Muhammad Yasir Khan, Hirra Iftikhar, Arslan Saleem Chughtai, Muhammad Hassan, Faisal Saud Dar, Irfan Ahmed

**Affiliations:** 1 Haider Ali Liver Transplant and Hepato-Pancreato-Biliary, Pakistan Kidney and Liver Institute and Research Center (PKLI & RC), Lahore, Pakistan; 2 Ihsan ul Haq Liver Transplant and Hepato-Pancreato-Biliary, Pakistan Kidney and Liver Institute and Research Center (PKLI & RC), Lahore, Pakistan; 3 Sohail Rashid Liver Transplant and Hepato-Pancreato-Biliary, Pakistan Kidney and Liver Institute and Research Center (PKLI & RC), Lahore, Pakistan; 4 Muhammad Yasir Khan Liver Transplant and Hepato-Pancreato-Biliary, Pakistan Kidney and Liver Institute and Research Center (PKLI & RC), Lahore, Pakistan; 5 Hirra Iftikhar Histopathology, Pakistan Kidney and Liver Institute and Research Center (PKLI & RC), Lahore, Pakistan; 6 Arslan Saleem Chughtai Research Center, Pakistan Kidney and Liver Institute and Research Center (PKLI & RC), Lahore, Pakistan; 7 Muhammad Hassan Research Center, Pakistan Kidney and Liver Institute and Research Center (PKLI & RC), Lahore, Pakistan; 8 Faisal Saud Dar Liver Transplant and Hepato-Pancreato-Biliary, Pakistan Kidney and Liver Institute and Research Center (PKLI & RC), Lahore, Pakistan; 9 Irfan Ahmed Research Center, Pakistan Kidney and Liver Institute and Research Center (PKLI & RC), Lahore, Pakistan

**Keywords:** Chronic cholecystitis, Gallbladder histology, Living liver donors, Transplantation

## Abstract

**Background & Objective:**

In low and middle-income countries (LMICs), living donor liver transplant (LDLT) is common due to limited cadaveric grafts. Cholecystectomy during donor hepatectomy allows evaluation of gallbladder pathologies in healthy donors. This study examined the prevalence of gallbladder pathologies in healthy living liver donors.

**Methodology:**

This study used retrospective data from March 2019 to March 2023 and prospective data from March 2023 to July 2024, collected from consecutive liver donors at a single liver transplant center using a prospectively maintained database. Gallbladders were classified as normal or abnormal based upon histopathology. Chi-square tests were used to determine associations between gallbladder pathologies with age, body mass index (BMI), blood group and Liver Attenuation Index (LAI).

**Results:**

Among 677 donors, 277 (40.92%) were female, with an age range of 18-53 years. None reported biliary symptoms during initial evaluation. Chronic cholecystitis was incidentally found in 68.69% of donors. Of these, 383 cases (82.37%) had chronic cholecystitis only, while 82 (17.63%) showed additional histopathological findings. Specifically, five cases (0.7%) had chronic cholecystitis with both cholesterolosis and gallstones, and 6 (0.9%) had it with gallstones only. Chronic cholecystitis showed a significant association with age (p < 0.001) and BMI (p = 0.018), but not with blood group (p = 0.121) or LAI (p = 0.086).

**Conclusion:**

Chronic cholecystitis is highly prevalent among asymptomatic living liver donors and is significantly associated with increasing age and BMI, this may raise concern about the health of the broader Pakistani population. These findings confirm that subclinical gallbladder pathology exists frequently in healthy individuals despite negative preoperative screening.

## INTRODUCTION

Since the first successful liver transplantation (LT) in 1967 by Starzl et al., LT is the standard definitive treatment option and a life-saving procedure for patients with chronic liver diseases with decompensation, liver cancers and a variety of metabolic liver diseases.^1^-^3^ In Western countries, the need for organs is primarily met by deceased donors, whereas in some countries like Pakistan, the focus is mainly on living donors.^2^-^4^

Given the gallbladder’s anatomical location, cholecystectomy is routinely performed during living donor hepatectomy (LDH) to evaluate the biliary tract’s structure and determine the demarcation line at the liver, an essential step in the donor hepatectomy.^5^,^6^ Gallbladder specimens removed during cholecystectomy are routinely sent for histopathology in accordance with the conventionally practiced standard.^7^,^8^ Although rare, this examination is crucial for identifying asymptomatic incidental pathologies in gallbladder specimens.^8^,^9^ This may provide valuable insights into the incidence and prevalence of various gallbladder pathologies in otherwise healthy adults.^10^ Among different gall bladder pathologies, chronic cholecystitis is the common gallbladder disease that is histopathologically identified during LDH.^11^

This study aimed to assess the prevalence of chronic cholecystitis and associated gallbladder pathologies in living donors who underwent hepatectomy in the local population. Additionally, it sought to evaluate the relationships between these pathologies and demographic factors such as age, gender, BMI, liver attenuation index (LAI) and blood group.

## METHODOLOGY

This study was conducted to evaluate the histopathological findings in the gallbladders of liver donors and assess the associations with several demographic parameters.

### Ethical Approval:

The Institutional Review Board (IRB) at PKLI&RC provided approval for this study with IRB No. (PKLI-IRB/AP/107-R; dated 09-03-2023), ensuring that all research protocols adhered to ethical standards and guidelines for the protection of human subjects. The IRB thoroughly reviewed the study’s design, data collection methods, and potential risks to participants before granting approval.

The study population consisted of all 700 healthy liver donors since start of the transplant programme at Pakistan Kidney and Liver Institute and Research Center (PKLI&RC), Lahore, Pakistan. Data was collected using a mixed design, with retrospective data from March 8, 2019 to March 5, 2023 and prospective data from 6 March 2023 to 10 July 2024. After excluding donors with missing or incomplete data, 677 donors (368 retrospective donors and 309 prospective donors) were included in the final analysis. The institutional criteria for living donor’s selection and liver biopsy criteria are summarized in Table-I.

**Table-I:** Selection criteria for donor evaluation.

Institutional criteria for Living Donor evaluation/selection;	Indication of liver biopsy in LLD:
A legally or genetically related person.	Presence of any two or more of the following factors.
Compatible blood group spouse.	BMI more than 30.
No major health problems.	Presence of steatosis in imaging studies:
Good physical and mental health.	USG abdomen: Grade I/II fatty liver.
Ages range from 18 - 50 years.	Fibro scan: S1/S2.
Altruistic donation	Contrast-enhanced CT Scan LAI <05.
Body mass index (BMI) range 18 — 30.	Blood tests show unusual results:
Must be free from habit or history of substance abuse.	Persistent deranged liver function test.
	Autoantibodies: ANA >1:80
	Hb core AB-positive donors. Strong family history of autoimmune disorders:
	Primary Biliary Cirrhosis (PBC).
	Primary Sclerosing Cholangitis (PSC).
	Autoimmune Hepatitis (AIH).
	Positive Genetic tests for:
	Alpha-1-antitrypsin deficiency.
	Hemochromatosis.

The gallbladder was received in a labelled container with patient identifiers and preserved in 10% formalin. The specimen was oriented, measured in three dimensions, and inked at the cystic duct and liver bed margins. Upon opening, gross features include the serosal and mucosal surfaces, wall thickness, presence and type of stones (if any), and any accompanying lymph nodes were documented. Representative sections were taken from the cystic duct margin and gallbladder wall (neck, body, and fundus) for histopathological evaluation. The corresponding blocks and slides from all living donor cholecystectomy cases were subsequently retrieved and reviewed by two consultant pathologists blindly.

After reviewing the slides, gallbladders specimen was classified as “normal” and “with pathological findings”. Cases “with pathological findings” were further categorized as “Chronic Cholecystitis only”, “Chronic cholecystitis with additional findings”. “Chronic cholecystitis with additional findings” included cases showing chronic cholecystitis with additional pathological findings such as cholelithiasis, cholesterolosis, pyloric metaplasia and ectopic pancreas. Further data on various other parameters were collected for each donor, including age, gender, blood group, body mass index (BMI), and LAI.

Age was recorded and categorized into three groups arbitrarily: under 28 years, 28-40 years, and over 40 years. Gender, blood group, including all major blood types along with Rh factors, and BMI noted. BMI was categorized into underweight, normal, overweight, obese. LAI was used to determine the presence of hepatic steatosis, categorizing donors into normal or hepatic steatosis groups based on non-contrast CT scan. LAI of 6 or more was considered normal.

### Statistical analysis:

All data were summarized in appropriate tables to facilitate comparison and interpretation using statistical software, SPSS 27. The Numerical data like age was presented in the form of Median and Range. Whereas categorical data like Gender, BMI, Blood Group and LAI etc. were presented in the form of frequencies and Percentages. Numerical data like age, body mass index (BMI), and Liver Attenuation Index (LAI) were transformed into categories using standard classification. Chi square test, and related special case test, was used to determine the association between chronic cholecystitis and each of the study variables. Binary logistic regression was fitted to predict Chronic cholecystitis. A p-value of less than or equal to 0.05 was considered statistically significant.

## RESULTS

**Fig.1:**
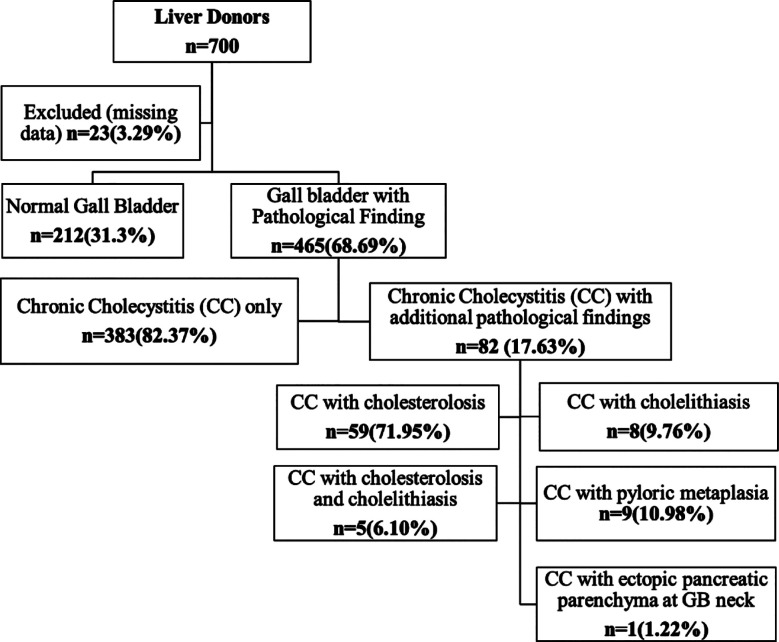
Tree diagram for distribution of chronic cholecystitis among living donors.

**Fig.2:**
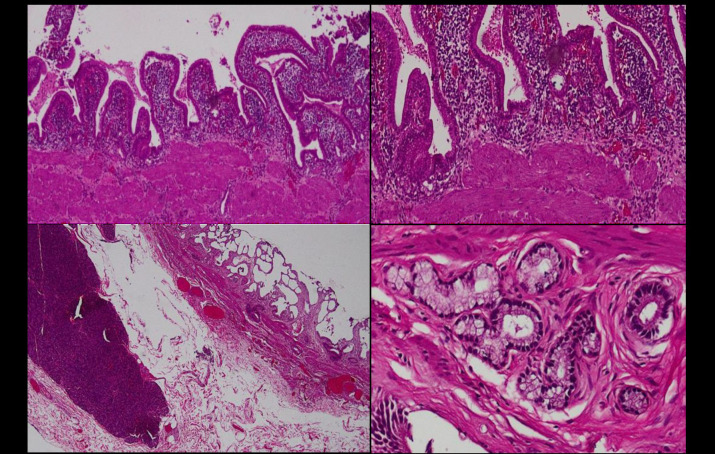
(A &B) Chronic cholecystitis with Rochitansky Aschoff sinus. C) Gall bladder with heterotopic pancreas. D) Gall bladder with pyloric gland metaplasia.

In this study, 677 gallbladders were included in the final analysis. Among the liver donors, 400 (59.1%) were male and 277 (40.9%) were female. The median age of male donors was 24 years (Range 18-53 years), while the median age of female donors was 26 years (range 18-48 years). Among the 677 donors, 212 (31.0%) exhibited normal gallbladder histology while chronic cholecystitis was identified in 465 (68.69%) gallbladders. Within this cohort, “chronic cholecystitis only” accounted for 383 (82.37%) cases, whereas “chronic cholecystitis with additional pathological findings” was documented in 82 (17.63%) cases. Fig.1 represents the frequency of additional findings in GB specimen on histology in the tree diagram. The histological appearance of some of the mentioned gallbladder findings is shown in Fig.2.

Of the total liver donor pool, 453 (66.9%) were young adults aged below 28, 194 (28.7%) were between 28 to 40 years old, and 30 (4.4%) were above 40. Notably, Age wise comparison reveals that chronic cholecystitis was present in 292(64.46%) of donors aged less than or 28 years, among 28-40 years it was present in 146(75.26%) and in more than 40 years it was present in 27(90.00%). A statistically significant association was found between age and chronic cholecystitis (p-value <0.001) as shown in Table-II.

**Table-II:** Comparison of cholecystitis regarding different parameters.

Study Variables	Categories	Chronic cholecystitis		Total	p-value
		Present n=465 (68.69%)	Absent n=212(31.31%)		
Age Group	<28	292(62.8%)	161(75.9%)	453(66.9%)	<0.001*
	28-40	146(31.4%)	48(22.6%)	194(28.7%)	
	>40	27(5.8%)	3(1.4%)	30(4.4%)	
Gender	Male	266(57.2%)	134(63.2%)	400(59.1%)	0.141
	Female	199(42.8%)	78(36.8%)	277(40.9%)	
Blood Group	O^+^	176(37.8%)	82(38.7%)	258(38.1%)	0.121
	O^-^	12(2.6%)	15(7.1%)	27(4.0%)	
	A^+^	88(18.9%)	46(21.7%)	134(19.8%)	
	A^-^	7(1.5%)	2(0.9%)	9(1.3%)	
	B^+^	154(33.1%)	59(27.8%)	213(31.5%)	
	B^-^	10(2.2%)	4(1.9%)	14(2.1%)	
	AB^+^	17(3.7%)	4(1.9%)	21(3.1%)	
	AB^-^	1(0.2%)	0(0.0%)	1(0.1%)	
BMI	Under Weight	21(4.5%)	17(8.0%)	38(5.6%)	0.018*
	Normal (18.5-24)	268(57.6%)	138(65.1%)	406(60.0%)	
	Overweight	162(34.8%)	54(25.5%)	216(31.9%)	
	Obese (30-35)	14(3.0%)	3(1.4%)	17(2.5%)	
Liver Attenuation Index (LAI)	Hepatic steatosis	118(25.4%)	41(19.3%)	159(23.5%)	0.086
	Normal	347(74.6%)	171(80.7%)	518(76.5%)	

Additionally, 258 (38.1%) participants had blood group O+, 27 (4.0%) had O-, 134 (19.8%) had A+, 9 (1.3%) had A-, 213 (31.5%) had B+, 14 (2.1%) had B-, 21 (3.1%) had AB+, and 1 (0.1%) had AB-. There was no significant association between blood group and chronic cholecystitis (p-value 0.121). The BMI classification revealed that 38 (5.6%) were underweight, 406 (60.0%) were in the normal range, 216 (31.9%) were overweight, 17 (2.5%) were obese. Among the donors with chronic cholecystitis (465, 68.69%), 21(4.5%) were underweight, 268(57.6%) were normal, 162(34.8%) were overweight and 14(3.0%) were obese. A significant association was found between chronic cholecystitis and BMI (p-value 0.018). Regarding the LAI, 159 (23.5%) donors had mild to moderate hepatic steatosis, while 518 (76.5%) were classified as normal, no liver donors were observed with severe hepatic steatosis. There was no significant association between chronic cholecystitis and LAI (p-value 0.086) as shown in Table-II.

As the frequency of chronic cholecystitis among donors was unexpectedly high, all the slides of gallbladder specimens were re-evaluated by the histopathology department to validate the results. Discrepancies were documented in only 12/677 (1.77%) cases. Out of 465 chronic cholecystitis, 10 (2.15%) were re-evaluated as normal. Among 383 cases of “chronic cholecystitis only” two cases showed additional finding of cholesterolosis. Thus overall, out of 677, 222(32.8%) donors have normal gallbladder and 455(67.2%) were having Chronic cholecystitis. Among the chronic cholecystitis cohort, 371(54.8%) were having “chronic cholecystitis only” and 84(12.4%) cases had “chronic cholecystitis with additional histological findings”.

The relative risk of chronic cholecystitis among donors older than 25 years was 1.76 (95% CI: 1.25–2.48). Compared with normal-weight donors, the relative risk of chronic cholecystitis was 0.66 (95% CI: 0.34–1.31) in underweight donors, 1.38 (95% CI: 0.95–2.02) in overweight donors, and 2.16 (95% CI: 0.60–7.72) in obese donors. Donors with blood group O positive (Rh-positive) had a relative risk of 2.94 (95% CI: 1.29–6.67). The baseline model included age, gender, BMI, LAI, blood group O positive, and Rh factor variables to predict chronic cholecystitis. The final model explained 5.6% of the variation.

## DISCUSSION

Research on histopathological changes in the gallbladder primarily relies on data from patients who have undergone cholecystectomy due to symptomatic/complicated gallbladder disease or malignancy. Secondly, it is not ethically feasible to perform cholecystectomy on healthy individuals without any clinical indication to obtain histopathological data.^12^ However, performing cholecystectomy as a routine procedure in living liver donors (LLDs) who are completely healthy, and subsequently evaluating the histopathological structure of the gallbladder specimens, provide valuable insights into the natural variations of gallbladder histology in healthy asymptomatic individuals. The findings from such studies could significantly enrich the existing literature.^10^

To date, limited literature has examined gallbladder specimens from healthy LLDs.^5^,^8^,^10^,^11^ This study aimed to provide a comprehensive update on gallbladder in our local healthy population and understanding of gallbladder histology in the context of liver donation.

The lifetime risk of developing gallstones varies from 10% to 20%, among them 80% of cases are asymptomatic.^13^ It is four times more common in women than in men, and this rate becomes equal as age progresses.^14^ Asymptomatic gallstones are a common pathology and are thought to be responsible for acute and chronic cholecystitis. In contrast, the current study reveals a significant prevalence of chronic cholecystitis without gall stones. The findings of the current study, validate published literature that highlights chronic cholecystitis as a common condition encountered during cholecystectomies performed in conjunction with living donor hepatectomy.^11^,^12^

The most significant finding of the current study is the unexpectedly high prevalence of chronic cholecystitis among asymptomatic living liver donors. In a cohort of 677 clinically healthy individuals aged 18–53 years, histological evidence of chronic cholecystitis was incidentally identified in 68.69% of subjects, despite the absence of symptoms. Detailed analysis revealed that the vast majority of these cases (82.37%) represented isolated chronic cholecystitis, while combinations with cholelithiasis (0.9%) or cholesterolosis were rare. Furthermore, this subclinical pathology demonstrated a strong statistical association with increasing age (p < 0.001) and Body Mass Index (p = 0.018) whereas no significant correlation was observed with blood group (p = 0.121) or Liver Attenuation Index (p = 0.086).

The routine histopathological examination of resected gallbladders is crucial, as it can uncover asymptomatic diseases, including early stage T1 gallbladder cancer which is diagnosed only on histopathology and might otherwise go undetected. In contrast to our findings, international studies report a much lower incidence of chronic cholecystitis in living donors, out of 2577 gallbladder histopathological analysis from Inonu University, Turky, 17.9% living donors had chronic cholecystitis and 10.44% of cases exhibited additional findings such as cholesterolosis in 8.3%, cholelithiasis in 1.38%, and pyloric metaplasia in 0.04% subjects.^12^ Moreover, a similar study conducted by Akbulut S, et al. shows that chronic cholecystitis was in 18.9%, cholesterolosis in 3.86%, cholelithiasis in 1.38%, and pyloric metaplasia in 0.10% of 1009 patients cohort.^10^ In a study conducted by Elsarawy et al., chronic cholecystitis was observed in 122(41%), out of 295 donors, either as a single condition or alongside other pathologies.^11^ In comparison to literature, our cohort demonstrated a much higher prevalence of chronic cholecystitis (68.69%), in which only 13 (2.80%) were associated with gallstones. In another study by Bhatti and colleagues from Pakistan, around 52% of donors’ gallbladders were diagnosed as chronic cholecystitis.^8^ These abnormal findings of high percentage of cholecystitis is an alarming finding for the Pakistani population. This elevated rate highlights the urgent need for increased awareness and screening for gallbladder diseases.

The disparity between our high prevalence rates and lower international figures and high prevalence of chronic cholecystitis observed in this asymptomatic cohort may be attributed to specific environmental and biological factors prevalent in South Asia. A regional study in the Gangetic basin of North India has identified environmental links, such as heavy metal contamination in water sources and specific dietary habits, which may predispose the local population to gallbladder pathology.^15^ Furthermore, metabolic risk factors, such as obesity and dyslipidemia, are well-established contributors to gallbladder disease.^16^ Although our study did not quantify these environmental exposures, the elevated BMI observed in our donors supports the hypothesis that metabolic factors may play a significant role.

In the current study, there is a significant association between chronic cholecystitis and age. While chronic cholecystitis has traditionally been viewed as a condition more prevalent in older populations, our findings indicate that a considerable number of younger donors also present with this pathology. This highlights the necessity for thorough preoperative screening of gallbladder in all age groups. Furthermore, a study by Bhatti et al. found that gallbladder pathology was more common in donors with a BMI greater than 25 kg/m², with a prevalence of 69.3% compared to 30.7% in those with a BMI of 25 kg/m² or less.^8^ Similarly, our findings align with Bhatti et al., showing that cases of chronic cholecystitis increase with rising BMI.

### Strength of Study:

The primary strength of this study lies in its use of healthy living liver donors, which provides a rare “gold standard” control group often missing in pathological research. In the context of Pakistan, where gallbladder carcinoma and lithiasis are significant health burdens, establishing a histological baseline for the “asymptomatic” population is critical. Most existing local literature relies on cholecystectomy specimens from symptomatic patients, which inherently biases findings toward advanced disease. By analyzing strictly screened donors, this study offers unique insights into the subclinical burden of gallbladder disease in the Pakistani population that routine clinical practice serves to obscure.

However, this single-center study is limited by the assessment of only demographic factors (age, gender, BMI), while other relevant variables such as genetic predisposition, lifestyle, geography, and dietary habits were not analyzed due to lack of data. While the study provides valuable insights into the prevalence of chronic cholecystitis, it does not establish causal relationships, which warrants more investigation through further prospective studies to validate the findings and explore the underlying mechanisms. We are planning to run further studies to investigate possible explanations for this finding.

To better understand the high prevalence of chronic cholecystitis in asymptomatic individuals, future research should focus on multicenter collaborations across Pakistan. This would help validate whether these findings are a nationwide phenomenon or specific to our catchment area. Additionally, prospective studies incorporating detailed lifestyle questionnaires are essential to identify specific risk factors, such as dietary patterns (particularly high-fat consumption) and environmental exposures (e.g., heavy metal contamination in water sources), which are hypothesized to contribute to gallbladder pathology in the region. Finally, molecular and genetic analyses are needed to elucidate whether the South Asian population possesses a distinct genetic predisposition to subclinical gallbladder inflammation compared to Western cohorts.

## CONCLUSION

This study highlights a significant prevalence of chronic cholecystitis among asymptomatic living liver donors, offering a unique histological baseline for the healthy Pakistani population. The strong association with age and BMI suggests that subclinical gallbladder inflammation is common even in rigorously screened individuals who lack clinical symptoms. Consequently, routine histopathological evaluation of the resected gallbladder remains essential to identify these incidental pathologies. Future prospective, multicenter studies are warranted to validate these findings across the region and investigate the environmental and metabolic drivers of this high prevalence.
